# Genetic Diversity, Evolutionary Dynamics, and Pathogenicity of Ferret Badger Rabies Virus Variants in Mainland China, 2008–2018

**DOI:** 10.3389/fmicb.2022.929202

**Published:** 2022-07-14

**Authors:** Faming Miao, Jinghui Zhao, Nan Li, Ye Liu, Teng Chen, Lijuan Mi, Jinjin Yang, Qi Chen, Fei Zhang, Jie Feng, Shunfei Li, Shoufeng Zhang, Rongliang Hu

**Affiliations:** ^1^Changchun Veterinary Research Institute, Chinese Academy of Agricultural Sciences, Changchun, China; ^2^Suzhou Shangfangshan Forest Zoo, Suzhou, China; ^3^Department of Innovative Medical Research, Chinese People’s Liberation Army General Hospital, Institute of Hospital Management, Beijing, China

**Keywords:** ferret badger, rabies virus, epidemiology and evolution, pathogenicity, neutralizing antibody

## Abstract

In contrast to dog-associated human rabies cases decline year by year due to the rabies vaccination coverage rates increase in China, ferret badger (FB, *Melogale moschata*)-associated human rabies cases emerged in the 1990s, and are now an increasingly recognized problem in southeast China. To investigate epidemiology, temporal evolution dynamics, transmission characterization, and pathogenicity of FB-associated rabies viruses (RABVs), from 2008 to 2018, we collected 3,622 FB brain samples in Jiangxi and Zhejiang Province, and detected 112 RABV isolates. Four FB-related lineages were identified by phylogenetic analysis (lineages A–D), the estimated Times to Most Recent Common Ancestor were 1941, 1990, 1937, and 1997 for lineages A–D, respectively. Furthermore, although no FB-associated human rabies case has been reported there apart from Wuyuan area, FB-RABV isolates are mainly distributed in Jiangxi Province. Pathogenicity of FB-RABVs was assessed using peripheral inoculation in mice and in beagles with masseter muscles, mortality-rates ranging from 20 to 100% in mice and 0 to 20% in beagles in the groups infected with the various isolates. Screening of sera from humans with FB bites and no post-exposure prophylaxis to rabies revealed that five of nine were positive for neutralizing antibodies of RABV. All the results above indicated that FB-RABV variants caused a lesser pathogenicity in mice, beagles, and even humans. Vaccination in mice suggests that inactivated vaccine or recombinant subunit vaccine products can be used to control FB-associated rabies, however, oral vaccines for stray dogs and wildlife need to be developed and licensed in China urgently.

## Introduction

Rabies is an acute, progressive, and encephalitic disease. Once clinical signs manifests, the disease usually is considered to be invariably fatal. It continues to present public health problems worldwide and causes more than 60,000 human deaths annually (Fooks et al., 2014). The majority of these deaths are in developing countries, primarily in Africa and Asia, more than 95% of human rabies cases are attributed to dog bites worldwide and the ratio is higher in developing countries (Taylor et al., 2017). However, in southeast China, the percentage of dog-associated human rabies is relatively low, up to 80% of the reported human rabies cases were inferred to be caused by ferret badger (FB) bites in some districts in Zhejiang and Jiangxi province (Gong et al., 2007; Wang et al., 2014).

The Chinese FB, which dwells mainly in southeastern China. These mustelids have several names like crab-eating mongoose, rice field dog, viviparid-eating dog, loach-eating dog, and white face weasel—mainly because of their omnivorous behavior and external appearance. FB-associated human rabies cases in China were emerged in 1994; 46 human rabies cases exposed to FB bites were reported in Zhejiang and Jiangxi province from 1994 to 2007.

In 2008, we isolated the first FB rabies virus strain, since then, we continued our surveillance, and collected additional positive samples mainly distributed in Jiangxi and some in Zhejiang province that were isolated from FB. We have conducted laboratory-based surveillance, virus isolation, nucleotide sequencing, and molecular characterization analysis (Zhang et al., 2009, 2010; Zhao et al., 2014a). Recently, we investigated the temporal dynamics of FB-associated rabies viruses (FB-RABVs) in China based on N sequences from samples collected from 2014 to 2016, results showed that almost all FB isolates until 2013 belonged to the China canine RABV Group II apart two isolated strains in the China canine RABV Group I. However, since 2014, all RABVs isolated from the FBs in Jiangxi have been classified under the China I canine RABV Group I (Miao et al., 2018). Further study demonstrated that the increasing genetic diversity of RABVs in FBs is a result of the selective pressure from coexisting dog rabies virus, and FB rabies is likely occurring as an independent enzootic that became established in the FB populations from a dog RABV variant distributed in southeast China (Liu et al., 2010), which served as a reservoir for human infections and a source of new variants capable of emerging or reemerging in the future (Shi et al., 1997); however, little is known about the genetic diversity of FB-associated RABVs, their evolutionary dynamics and the role of FBs in maintaining RABV transmission in the epizootic regions, especially the pathogenicity of FB RABV remains to be unclear.

In this study, we collected 3,622 FB brain samples from 2008 through 2018 in these regions and detected 112 FB-RABV isolates from the collected samples, and sequenced the full-length of the nucleoprotein (N) gene from each isolate, constructed the phylogenetic and temporal evolution dynamics analysis. Furthermore, we determined the pathogenic characteristics of FB RABV in mice and beagles, tested the serum antibody titers for RABV who bit by FBs. Moreover, we reported that using inactivated vaccines or adenovirus recombinant vaccines can protect mice from lethal challenges with FB RABV infection.

## Materials and Methods

### Ethics Statement

All animal experiments described in this paper have been conducted according to the Guideline on the Humane Treatment of Laboratory Animals stipulated by the Ministry of Science and Technology of the People’s Republic of China (MOST) and approved by the Animal Welfare Committee of the Changchun Veterinary Research Institute, China. All animals were housed in a climate-controlled laboratory with a 12 h day/12 h night cycle.

Mice were purchased from Changchun Institute of Biological Products, China. Healthy, non-rabies vaccinated, 5-month-old healthy female beagles were obtained from Bolong experimental animal Co., Ltd., Qingdao, China. All dogs were housed individually in temperature- and light-controlled quarters in the Animal Facility, Jilin University.

### Sample Collection

**FIGURE 1:**
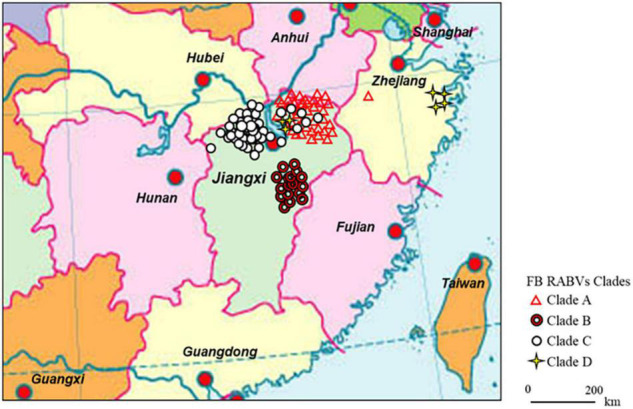
Continental distribution of ferret badger (FB) rabies virus (RABV) isolates in southeast China during 2008–2018. The 41 FB RABV isolates in Lineage A were mainly distributed in Qiandao lake (Zhejiang province) and east Poyang lake (Jiangxi province) regions. Lineage B had 17 isolates from Fuzhou district, south of Poyang lake. Lineage C included 47 isolates, 41 distributed in west Poyang lake, and the other 6 in east Poyang lake, mixing geographically with Lineage A. Lineage D had 4 isolates in Taizhou district, Zhejiang province and 3 in east Poyang lake.

From May 2008 through 2018, 3,622 FB brain tissue samples were collected across the Poyang lake (Jiangxi province) and Qiandao lake regions (Zhejiang province) (Figure 1). Most of the samples were obtained from the Poyang lake Region, because this is the most densely inhabited FB area in China. Both dead and living FBs were collected from the field and their heads (after euthanization if still alive) were shipped to our laboratory for rabies detection and RABV isolation.

### RNA Extraction, RT-PCR, and Sequencing

Total RNA of the collected FB brain homogenate was extracted by a single-step method using Trizol Reagent (Invitrogen, Life Technologies, United States) according to the manufacturer’s instructions. For nucleic detection, One-step reverse transcription (RT)-polymerase chain reaction (PCR) was used to obtain the lyssavirus sequences as previously described (Liu et al., 2013). As for RABV positive template RNA, one-step RT-PCR was performed with the designed primer sets according to the nucleoprotein (N) gene sequence of FB-RABV JX08-45 (GenBank: GU647092), primers cover the ORF of N gene:

N-F (1-22): 5′-ACGCTTAACAACAAAATCAGAG-3′;
N-R (1519-1540): 5′-CTCGGATTTACGAAGATCTTGC-3′.

The PCR conditions used were: reverse reaction at 50°C for 30 min; denaturation at 94°C for 2 min, then 35 cycles at 94°C for 30 s, 52°C for 30 s, and extended at 72°C for 1 min 30 s, followed by a final extension at 72°C for 7 min. After 1% agarose gel electrophoresis, the expected PCR bands were purified, then the products were sequenced twice using the N-F/R primers (Sangon Biotech Co., Ltd., Shanghai, China).

### Phylogenetic Analysis

A dataset of 244 complete N gene sequences from China RABVs was compiled (Supplementary Table). Similarity scores and percentage identities of the sequences were calculated using the software DNAStar. Maximum Likelihood (ML) analysis was performed using MEGA version 8.0 (Kumar et al., 2008). Bootstrap support was estimated using 1,000 replicates.

### Evolutionary Analysis

The Bayesian Markov Chain Monte Carlo (MCMC) method in the BEAST v1.7.5 package^1^ was used to reconstruct the Maximum Clade Credibility (MCC) phylogenetic tree, estimate the nucleotide substitution rate (per site, per year), and calculate the Time of the Most Recent Common Ancestor (TMRCA) after sequence alignment (Drummond and Rambaut, 2007; Suchard et al., 2018). Mutation rate was determined under a relaxed uncorrelated lognormal molecular clock model using the general time reversible (GTR) substitution model, incorporating gamma distribution to include in the estimations of the variation rate among sites (Γ4) and invariable sites (I). The nucleotide substitution model that best fitted the dataset was determined by the jModelTest (Posada, 2008). All chains were run for 50 million generations to ensure sufficient mixing. Parameters and trees were recorded every 10,000 steps. An effective sample size (ESS) > 200 was achieved for all estimated parameters. The highest posterior densities (HPD) were calculated with 10% burn-in and checked for convergence using Tracer (v1.5).

### Virus Isolation (Mouse Inoculation Test)

Ferret badger brain tissues testing positive for rabies by RT-PCR were homogenized as a 10% suspension in Dulbecco’s modified Eagle’s medium (DMEM; Gibco, CA, United States) supplemented with antibiotics (100 IU/ml penicillin and 100 μg/ml streptomycin). The supernatant fluids were clarified by 8,000 × g for 10 min and aliquots of 25 μl were injected intracerebrally into 3-day-old suckling mice (Balb/c mice, Changchun Institute of Biological Products, China) according to an approved animal protocol (Koprowski, 1996). Animals were observed daily for 21 days post-injection and those showing symptoms of rabies were euthanized. Brain tissues were removed and tested for RABV antigen by the direct fluorescent antibody (DFA).

### Pathogenicity of Ferret Badger-Rabies Viruses Isolates in Mice and Beagles

Eight FB-RABV strains used in this experiment (JX08-45, JX13-189, JX09-17, JX13-417, JX10-37, JX13-228, ZJ12-03, and ZJ13-431) were isolated in Jiangxi (Jingdezhen, Fuzhou, and Xiushui) and Zhejiang (Taizhou) province. RABV BD06 (GenBank accession no. EU549783.1) was isolated in our laboratory in 2006 from a rabid dog in China. This virus is maintained in dogs and it was shown in our previous experiments to cause 80% mortality in unvaccinated dogs after intramuscular injection. In addition, this virus strain is responsible for the majority of rabies cases among humans and dogs in China (Zhao et al., 2014b). All FB-RABV isolates were from brain tissues and were passaged three times intracerebrally in 3-day-old suckling mice (Balb/c mice, Changchun Institute of Biological Products, China). When moribund, the mice were euthanized. A total of 10% (w/v) suspensions were homogenized in DMEM containing 10% newborn calf serum and antibiotics, centrifuged to remove debris, and the supernatants were stored at −80°C.

Titers of the viral isolates were determined by intracerebral (IC) injection of 10-fold dilutions into 5-week-old female Balb/c mice, and median mouse IC lethal doses (MICLD_50_) were calculated using the Reed-Muench formula. Five 5-week-old female Balb/c mice per group were intramuscularly (IM) inoculated with 0.1 ml of 10^4^ MICLD_50_ of each strain into the left thigh muscle. Control animals were mock infected with 0.1 ml of DMEM containing 10% calf serum and antibiotics. The mice were observed twice daily for 28 days post inoculation (dpi): early morning, when body weights were measured, and late afternoon. Incubation period was taken as the number of days from injection to first appearance of neurological symptoms such as tremors or paralysis of one hind leg. In cases when death was imminent in the late afternoon, animals were euthanized and the morbidity period was taken as being the next day.

For further evaluate the pathogenicity in dogs, four groups (JX13-189, JX13-228, ZJ13-431, and BD06) of female beagles (*n* = 5) were inoculated IM into both left and right masseter muscles. Each inoculated animal received a total of 10^4^.^4^ MICLD_50_ of each viral isolate. Control animals were injected with DMEM containing 10% calf serum and antibiotics. Ninety days postinoculation (PI) all surviving animals were challenged with rabies virus (canine street virus; 1 × 10^5^.^4^ MICLD_50_) by the inoculation of 0.5 ml each in the left and right masseter muscles. The brainstem was collected at necropsy and tested by DFA. After challenge, the beagles were observed for another 90 days, and all surviving animals were euthanized to collect brain samples and tested by DFA at the end of the experiment.

### Serological Survey Among Humans Who Exposure to Ferret Badger Rabies Viruses

Nine sera were obtained from the volunteers collected during an investigation of humans bitten by FB more than twice during the 3 years and with no rabies post-exposure prophylaxis (PEP) treatment. All participants involved in the study had written informed consent. Sera were separated by centrifugation following incubation at ambient temperature for 3 h, inactivated at 56°C for 30 min; each sample was tested for rabies virus-specific neutralizing antibodies (VNA) by the standard fluorescent antibody virus neutralization (FAVN) test using BHK-21 cells (Cliquet et al., 1998).

### Rabies Vaccination in Mice and Challenge With Ferret Badger Rabies Virus

To determine the availability of emergency prophylaxis to the exposure to FB rabies, 25 Balb/c mice were randomly divided into 5 groups and immunized IM with a single injection 100 μl (containing NIH potency 0.2 IU) of canine inactivated vaccine produced by Jilin Heyuan Bioengineering Co. Ltd., human inactivated vaccine produced by Liaoning Chengda Bioengineering Co. Ltd., and other vaccination produced by our lab (attenuated SRV9 and a recombinant Canine Adenovirus Vaccine CAV-2-E3D-RGP), the placebo group was injected with DMEM as control. After 14 days post vaccination, mice were challenged with 0.1 ml of 10^4^ MICLD_50_ of FB-JX08-45 into the left thigh muscle, and housed for another 28 days, all survival mice were sacrificed after serum collection. Sera were separated and rabies virus neutralizing antibody (RVNA) was assayed using the standard FAVN method at 7 and 14 days post vaccination.

#### Accession Numbers

The new gene sequences in this study were submitted to NCBI GenBank nucleotide sequence database, accession numbers supplied as Supplementary Table.

## Results

### Ferret Badger Rabies Viruses Were Constantly Detected Over the Years in Poyang and Qiandao Lake Regions

As depicted in Table 1, from 2008 to 2018, FB-associated RABV nucleic acids were regularly detected in our collected samples from the Poyang lake region, with a positive rate ranging from 1.7 to 6.3%. In the Qiandao lake region, we detected 5 RABV positive among 138 samples from 2008 through 2013 (Table 1). A total of 112 FB RABV strains were detected and 70% infectious viruses were successfully isolated from all positive tissues and cultured in suckling mouse brain. The full-length of the N gene from each positive sample was amplified by RT-PCR and sequenced for phylogenetic and evolutionary studies.

**TABLE 1:** Rabies virus detection in ferret badgers in Poyang and Qiandao lake regions, 2008–2018.

Year	Positive numbers/Total tested numbers (% positive)		
	Poyang Lake	Qiandao Lake	Total
2008	4/63(6.3)	1/6 (16.7)	5/69(7.2)
2009	10/294(3.4)	0/63	10/357(2.8)
2010	4/148(2.7)	0/9	4/157(2.5)
2011	6/248(2.4)	0/20	6/268(2.2)
2012	13/751(1.7)	1/19 (5.2)	14/770(1.8)
2013	24/479(5.0)	3/21 (14.2)	27/500(5.4)
2014	4/126(3.2)	/	4/126(3.2)
2015	1/36(2.8)	/	1/36(2.8)
2016	10/363(2.8)	/	10/363(2.8)
2017	24/677(3.5)	/	24/677(3.5)
2018	7/299(2.3)	/	7/299(2.3)
Total	107/3484(3.1)	5/138 (3.6)	112/3622(3.1)

### Segregation of Ferret Badger Rabies Viruses Into Geographic Lineages and Evidence of Rabies Viruses Cross Species Transmission Between Ferret Badgers and Dogs

**FIGURE 2:**
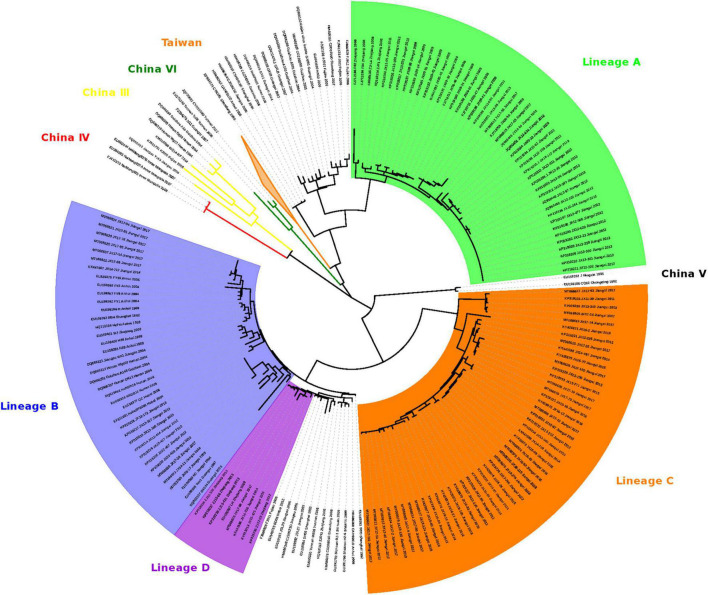
Maximum likelihood phylogeny of 244 RABV sequences separates the 112 FB-associated RABVs into 4 distinct Lineages: A, B, C, and D. Lineage A was rooted with China dog RABV Group II, Lineages B and D with China dog RABV Group I, and Lineage C was independent, sitting in the middle of Group I and II. The numbers below the branches were bootstrap values (%) of 1,000 replicates.

The 112 FB-associated RABVs were classified into four lineages (A–D) using data from 132 Chinese RABV N gene sequences (Figure 2), and each lineage was grouped geographically by the location of the virus isolate (Figure 1). Lineage A, with 41 FB-associated RABV isolates, 40 distributed in east of Poyang lake, and 1 distributed in Zhejiang province, was embedded in China canine RABV Group II (China II). The FB RABVs in lineage A were closely related to Chinese vaccine strains CTN181 and CTN7, which were derived from a dog street virus in the 1950s (Du et al., 2008).

Lineages B, C, and D were grouped with China canine RABV Group I (China I) (Guo et al., 2013). Lineage B had 17 isolates from the Fuzhou district, south of Poyang Lake (Zhang et al., 2010). Lineage D had 7 FB isolates, 4 FB isolates (ZJ12-03, ZJ13-66, ZJ13-130, and ZJ13-431) are located in Taizhou district, Zhejiang province and other 3 isolates are located in east Poyang lake. Of note, a canine RABV isolate is denoted as LH from Zhejiang province, where Qiandao lake is located, was embedded in the FB lineage D, and lineage D and canine RABV Group are closely related phylogenetically. Lineage C included 47 FB-associated RABVs, from 2008 to 2018, 41 isolated from west Poyang lake, and the other 6 from east Poyang lake, mixing geographically with Lineage A (Figure 1). However, lineages C and A were phylogenetically distinctly separate. The 47 isolates within Lineage C, the majority located in the west of Poyang lake, shared approximately 95% nucleotide identity with China canine RABV Group I, and 88% nucleotide identity with China canine RABV Group II.

### The Time of Ferret Badger Rabies Viruses Emergence in China by Evolutionary Analysis

The Bayesian MCMC approach used assumed a constant population size and an uncorrelated log normal molecular clock. The mean rate of nucleotide substitution for the N gene was 5.19 × 10^–4^ substitutions per site per year (95% HPD values, 3.48–7.05 × 10^–4^) which is close to that reported previously (Bourhy et al., 2008; Talbi et al., 2009). The calculated the Time of the Most Recent Common Ancestor (TMRCA) of China RABVs was 1745 CE (95% HPD values, 1700–1863). For FB Lineage A, the TMRCA was 1941 (95% HPD 41–100 years or year 1910–1969). For Lineage B, the TMRCA was 1990 (95% HPD 12–28 years or year 1982–1998). For Lineage C, the TMRCA was 1937 (95% HPD 42–104 years or year 1906–1968). For Lineage D, the TMRCA was 1997 (95% HPD 9–18 years or year 1992–2001). Lineages B and D could be recent events diverged from dog RABV spillovers to the FB populations, while lineages A and C could represent old historical spillovers with subsequent establishment in the FB population. In our 3,622 FB samples, the chance of detecting a FB RABV isolate in Lineages A and C was 3.7-fold higher than finding a RABV in Lineages B and D (88 vs. 24 in the 3,622 samples).

### Pathogenicity of Ferret Badger-Rabies Viruses Isolates in Mice and Beagles

Following intramuscular (IM) inoculation of 5-week-old mice, all mice infected with the JX08-45, JX13-189 and BD06 strains died within 6–11 dpi after showing typical neurological symptoms like the appearance of tremble, shaking, anger, then later hind limb paralysis of one or both limbs. However, the severities of infection were clearly different in other isolates (Table 2). The weight loss occurred during the disease progression from the onset of clinical signs to the point of death. Infected mice lost on average 19.3–32% of their overall body weight during the disease progression (data not shown). The incubation periods of the isolates varied from 4 to 12 days: JX13-189 had the shortest incubation period; mice infected with JX13-417 and ZJ12-03 had nearly the same incubation periods and clinical signs; and JX13-228 and JX10-37 infected mice had the longest incubation period (Table 2).

**TABLE 2:** Morbidity, incubation, and morbidity period of mice following intramuscular inoculation with each strain.

Strain	Lineage	Fatality/inoculated	Incubation period (days)		Mean morbidity period (days)
			r	Mean	
BD06	China I	10/10	6–8	6.6	1.2
JX13-189	FB-Lineage A	10/10	3–5	4	2
JX08-45	FB-Lineage A	10/10	4–7	6	2.6
JX13-417	FB-Lineage B	6/10	7–13	10.7	2.3
JX09-17	FB-Lineage B	6/10	5–8	6.7	3.7
JX13-228	FB-Lineage C	2/10	12	12	6
JX10-37	FB-Lineage C	6/10	11–13	12	6
ZJ12-03	FB-Lineage D	8/10	7–11	9.8	2.2
ZJ13-431	FB-Lineage D	8/10	5–6	5.8	2

To further evaluate their pathogenicity in dogs, four groups of female beagles (*n* = 5) were inoculated IM into masseter muscles, all animals were observed for 90 days post-inoculation. The mortality-rate of Zhejiang isolates, ZJ13-431, was 20% (1/5). Other 2 FB-RABV isolates, JX13-189 and JX13-228, exhibited no pathogenicity in beagles. Six of fifteen survived animals had detectable virus neutralizing antibodies (VNA) for rabies (0.13–2.6 IU/ml). After challenge with lethal BD06, rabies VNA of all the surviving animals had seroconverted (0.29–53.3 IU/ml) (Table 3).

**TABLE 3:** Rabies virus neutralizing antibody titers (IU/ml) and mortality of beagles post infection (p.i.).

Group	Fatality/Total	Incubation period (days)	VNAs (IU/mL) at days p.i.		After challenge
			0	30	14 days
BD06	4/5	9–16	0	0	53.3
ZJ13-431	1/5	19	0	0	1.5
			0	0	13.5
			0	0.29	40.5
			0	0	4.5
JX13-189	0/5	—	0	0.29	1.97
			0	0	0.87
			0	0.13	1.5
			0	0	1.14
			0	2.6	5.92
JX13-228	0/5	—	0	0	0.5
			0	0	40.5
			0	2.6	2.6
			0	0	0.29
			0	0.29	4.5

### Rabies Virus Neutralizing Antibodies Was Detectable in People With Frequent Contacts of Ferret Badgers

The nine human volunteers were selected from persons with frequent contacts with FB of more than 3 years’ duration. Testing of their sera revealed that 3 of the 9 had high titers of rabies virus–neutralizing antibodies (>2.6 IU/ml), and another two persons generated a certain amount of VNA titers (Table 4).

**TABLE 4:** Results of serologic rabies virus neutralizing antibody titers (IU/ml) in nine volunteers from Jiangxi province.

Volunteer No.	Area	Sex	Age (Year)	Time of bites	Rabies virus neutralizing antibodies (IU/mL)
H1	Fengxin County, Nanchang	M	47	Multiple	2.6
H2	Gaoan County, Jiujiang	F	47	Multiple	0
H3	Anyi County, Nanchang	M	40	Multiple	17.77
H4	Anyi County, Nanchang	M	62	Multiple	0.29
H5	Yongxiu County, Yichun	M	50	Multiple	0
H6	Nanchang	M	26	Multiple	10.26
H7	Hukou County, Jiujiang	M	46	Multiple	0.17
H8	Nanchang	M	47	Twice	0
H9	Jinxian County, Nanchang	M	60	Multiple	0

### Rabies Virus Neutralizing Antibody Test After Vaccination and the Survival Rate in Mice

**FIGURE 3:**
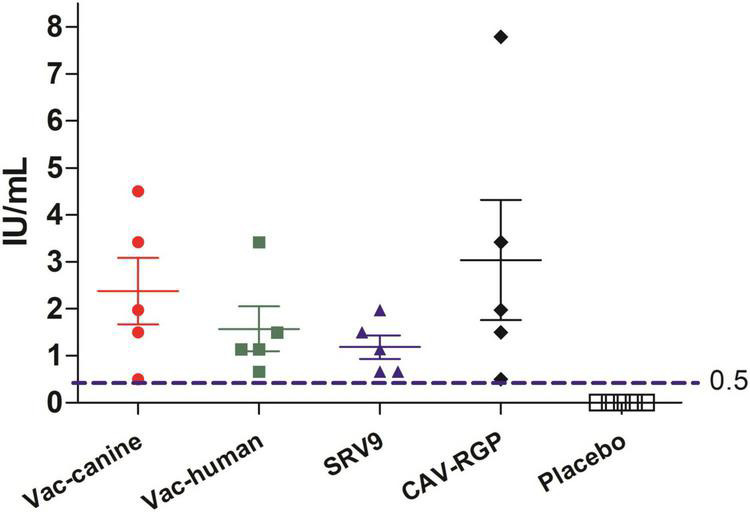
Rabies virus neutralizing antibody (RVNA) titers after 14 days vaccination in mice.

The cut-off value of rabies titer, 0.5 IU/ml, the minimum defined acceptable threshold as recommended for complete protection against rabies (Cliquet et al., 1998). No rabies VNA was detected in placebo group mice. In contrast, vaccinated mice had a small amount of rabies VNA titers after 7 days post a single vaccine injection, and after 14 days, rabies VNA titers of all vaccinated mice reach ≥ 0.5 IU/ml (Figure 3). After challenge with the FB-RABV, all mice had no obvious body weight change, and all survived, however, in the placebo treatment group, mice all developed rabies clinical symptoms and succumbed to death.

## Discussion

Rabies virus can infect a range of wildlife in specific geographic areas: foxes and raccoon dogs in Europe; foxes in the Middle East; raccoon dogs, camels, and FBs in Asia; skunks, foxes, coyotes, and mongooses in North America (Nadin-Davis and Fehlner-Gardiner, 2019); and mongooses in Africa (Mansfield et al., 2006; Vos et al., 2009; Liu et al., 2016; Troupin et al., 2016). Wildlife-associated rabies cannot be neglected in a rabies-free country, such as America, where the majority of human rabies is caused by bat bites (Patyk et al., 2012). In Taiwan, an outbreak occurred among FBs during 2012–2013 and a five-decade history of rabies-free humans and domestic animals ended, causing a public health threat (Chiou et al., 2014). In mainland China, where rabid dogs serve as the major source of infection, however, with the increase of vaccination coverage rates to domestic animals and enforcement to the stray dogs, dog-associated human rabies cases rapidly decrease, the wildlife-associated human rabies deaths are now an increasingly recognized problem in southeast China (Wang et al., 2014), especially that there is no vaccination or any other intervention to prevent FB-related RABV transmission in the animal population, which needs to investigate how RABV was introduced and maintained in the FBs urgently.

The retrospective investigation has revealed that FB-associated rabies was first reported in 1997; however, it first arose in 1994, and it has caused nearly hundreds of human fatalities, though this is likely an underestimated number. In this study, we focused on the Poyang and Qiandao lake regions where both human and dog rabies co-exists. For the Qiandao lake, FB RABV was only isolated in years 2008, 2012, and 2013. The absence of FB RABV detection from 2009 to 2011 was possibly due to inadequate surveillance. Interestingly, in our 112 FB RABVs positive from the 3,622 samples, 88 were in Lineages A and C: the lineages diverged in 1937 and 1941, respectively. Only 24 isolates were recovered for Lineages B and D, which we estimate as having diverged in 1990 and 1997. We speculate that our better chance (3.7 times) of finding a FB-associated RABV in lineages A and C is because the virus has been circulating for a longer time and is, therefore, better adapted in the FB population, which might explain, but not proven, its sustained transmission in the Poyang and Qiandao lake regions. Lineages B (year 1990) and D (1997) are corresponding with two major rabies epidemics in China (1980–1990 and 1997–present). Lineages A and C diverged from dog RABVs in the 1930s and 1940s when there was no national disease reporting system. However, TMRCA of China canine RABVs is estimated to be about 1745 CE in our calculation and other studies, suggesting RABV circulation in the FB population occurred after the emergence of the dog rabies virus (Tao et al., 2013; Troupin et al., 2016).

Numerous studies have shown that various wildlife can be colonized by dog RABV variants to become successful rabies host. Typical examples include the cases in Europe where rabies evolved from domestic dogs to raccoon dogs and foxes (Bourhy et al., 1999), the emergence of fox rabies in Colombia and Brazil (Bernardi et al., 2005; Paez et al., 2005; Carnieli et al., 2008), and wildlife rabies in the United States (Guerra et al., 2003). Other spillover events of rabies from dogs to wildlife have been observed in southern Africa (Nel et al., 1997; Sabeta et al., 2003, 2007; Zulu et al., 2009), Puerto Rico (Krebs et al., 2003; Nadin-Davis et al., 2008), and Turkey (Johnson et al., 2003). Because wildlife RABV variants can also transmit to unvaccinated dogs, thus entering urban environments, as has been observed in former Yugoslavia (Stankov, 2001). Our study supports the hypothesis that the rabies epizootic in the FB population is a consequence of dog RABV spillover at different times through multiple introductions. Rabies may have been circulating in the FB population for over 60 years (1937) before the first human death was reported in the 1990s. China’s priority for rabies control should focus on dogs; a complicating factor that needs to be considered is rabies in wildlife. It is noteworthy in our study that a canine RABV isolate from Zhejiang province was classified into FB Lineage D, which is composed of FB-RABV isolates (Figure 2). This could represent a cross-species transmission event in which either FBs introduced RABV to the dog population or dogs introduced RABV to FBs. Further investigation is needed when more RABVs are isolated from both animal species in the same region.

For rabies virus, selective pressures such as adaptation in a new host species favor the emergence of mutants with viral infectivity and pathogenicity change (Matsumoto et al., 2011). During the period when rabies virus adapted in FB, our results demonstrated that the pathogenicity of FB-RABV variants decreased to humans and other animals. The laboratory studies were conducted in mice and dogs, mortality-rates ranging from 20 to 100% in mice and 0 to 20% in beagles in the groups given the various isolates, which may indicate that FB-RABV variants can induce inapparent infections in mice and dogs. Intriguingly, some survival dogs developed almost less or no VNA in FB-RABV infection could protected from RABV challenging, however, the pathogenesis remains to be elucidated. Previously serological survey of FBs in southeast China found that 69.6% of 56 animals had serum neutralizing antibodies of rabies virus compared to 18.2% (14/77) seroconversion rate in unvaccinated dogs awareness by the owners, the higher seroconversion in the FBs is an interesting phenomenon, which may be due to inapparent infections with rabies virus in the FB population (Liu et al., 2010).

Evidence from laboratory and field studies suggested that inapparent rabies infection may occur in animals (Doege and Northrop, 1974). It has been suggested that the RABV strain causing enzootic rabies in Ontario in 1950s is a variant with low pathogenicity in humans and dogs (Filejski, 2016). No further study to provide an explanation for the phenomenon, so the evidence for inapparent rabies infection in humans was indefinite (Ruegsegger et al., 1961; Doege and Northrop, 1974). The explanation is that the persons who were bitten by rabid animals should receive PEP for rabies, thus the inapparent infection were covered up. In this study, the human volunteers of Jiangxi province were selected from the people who frequently contacted with FB and the duration was more than 3 years. We found nine persons, who participated in the survey, had been bitten by FB and never received rabies vaccines, 5 had detectable rabies neutralizing antibodies with 3 having protective level of antibody titers (≥2.6 IU/ml). This is the first definite evidence that the five human volunteers have been exposed to rabies virus through FB bites, and experienced an inapparent rabies infection without illness. Further studies are needed to proof whether these human subjects had inapparent rabies infection.

Nowadays, dog-associated human rabies cases decline rapidly with sufficient production levels for rabies vaccination of domestic dogs and PEP administrations enhanced, FB-associated rabies becomes to be a major threaten factor in some areas of China (Miao et al., 2021). Our study previously study showed that all FB had seroconverted serum, and 80% of the FBs with VNA titers ≥ 0.5 IU/ml either for oral immunization with a recombinant canine adenovirus or SRV9 (Zhao et al., 2014a). In this study, we demonstrate that only one dose vaccine either inactivated vaccine, attenuated vaccine or recombined subunit vaccine with intramuscular injection can be used to protect mice from lethal FB rabies virus challenging.

In conclusion, our study in the Poyang and Qiandao lake regions for the last 11 years suggests that RABV can be maintained in the FB populations without human intervention. The TMRCA of FB-associated rabies coordinates with canine rabies epidemics in China, and rabies in FBs is a later event after multiple introductions from the canine rabies virus. Rabies control in FBs poses a serious immediate concern in China. The potential of FB becoming an animal model, and the viral susceptibility of FB-RABVs to other animals, and the mechanism of inapparent infection needs further study. Together, Investigation of the phylogenetic and evolution of FB-associated rabies virus strains over the course of an epidemic can help in the development of strategies to combat and control viral disease (Badrane and Tordo, 2001; Pybus and Rambaut, 2009). Further, more research should be devoted to the development of oral vaccines for stray dogs and FBs.

## Data Availability Statement

The datasets presented in this study can be found in online repositories. The names of the repository/repositories and accession number(s) can be found in the article/Supplementary Material.

## Ethics Statement

The animal study was reviewed and approved by Changchun Veterinary Research Institute, Chinese Academy of Agricultural Sciences.

## Author Contributions

SZ and RH designed the experiments. FM, JZ, NL, YL, TC, LM, JY, and FZ performed the experiments. SL and QC analyzed the data. JF provided the advice. FM, JZ, SZ, and RH wrote the manuscript. All authors contributed to the article and approved the submitted version.

## Conflict of Interest

The authors declare that the research was conducted in the absence of any commercial or financial relationships that could be construed as a potential conflict of interest.

## Publisher’s Note

All claims expressed in this article are solely those of the authors and do not necessarily represent those of their affiliated organizations, or those of the publisher, the editors and the reviewers. Any product that may be evaluated in this article, or claim that may be made by its manufacturer, is not guaranteed or endorsed by the publisher.

## Footnotes

1
http://beast.bio.ed.ac.uk
