# Supplementary material for: Genetic Diversity, Evolutionary Dynamics, and Pathogenicity of Ferret Badger Rabies Virus Variants in Mainland China, 2008–2018
Source: Front Microbiol. 2022 Jul 14;13:929202. doi: 10.3389/fmicb.2022.929202 (PMC9330412; doi:10.3389/fmicb.2022.929202)
Supplement: Supplementary file 1 [file Data_Sheet_1.PDF]

Supporting information

S1 Table. The 244 China rabies virus data set, including 112 ferret badger associated rabies viruses in this study (isolated by our lab) and 59 FB rabies virus isolated from Taiwan.

NO.	Isolate	Origin	Host	Collection date	GenBank no.
1	JX08-45	Jiangxi	Ferret badger	2008	GU647092
2	JX08-48	Jiangxi	Ferret badger	2008	FJ719753
3	JX08-58	Jiangxi	Ferret badger	2008	FJ719755
4	JX08-47	Jiangxi	Ferret badger	2008	FJ719751
5	ZJ-LA	Zhejiang	Ferret badger	2008	FJ598135
6	JX09-18	Jiangxi	Ferret badger	2009	KP319184
7	JX09-55	Jiangxi	Ferret badger	2009	KP319185
8	JX09-41	Jiangxi	Ferret badger	2009	KP319186
9	JX09-08	Jiangxi	Ferret badger	2009	KP319187
10	JX09-01	Jiangxi	Ferret badger	2009	KP319188
11	JX09-14	Jiangxi	Ferret badger	2009	KP319189
12	JX09-24	Jiangxi	Ferret badger	2009	KP319190
13	JX09-39	Jiangxi	Ferret badger	2009	KP319191
14	JX09-42	Jiangxi	Ferret badger	2009	KP319192
15	JX09-17	Jiangxi	Ferret badger	2009	GU233765
16	JX10-36	Jiangxi	Ferret badger	2010	KP319221
17	JX10-37	Jiangxi	Ferret badger	2010	KP319222
18	JX10-67	Jiangxi	Ferret badger	2010	KP319224
19	JX10-66	Jiangxi	Ferret badger	2010	KP319223
20	JX11-05	Jiangxi	Ferret badger	2011	KP319193
21	JX11-51	Jiangxi	Ferret badger	2011	KP319194
22	JX11-109	Jiangxi	Ferret badger	2011	KP319195
23	JX11-30	Jiangxi	Ferret badger	2011	KP319225
24	JX11-56	Jiangxi	Ferret badger	2011	KP319226
25	JX11-139	Jiangxi	Ferret badger	2011	KP319227
26	JX12-64	Jiangxi	Ferret badger	2012	JQ950446
27	JX12-67	Jiangxi	Ferret badger	2012	JQ950448
28	JX12-102	Jiangxi	Ferret badger	2012	JQ950450
29	JX12-234	Jiangxi	Ferret badger	2012	JQ950452
30	JX12-244	Jiangxi	Ferret badger	2012	KP319196
31	JX12-477	Jiangxi	Ferret badger	2012	KP319197
32	JX12-566	Jiangxi	Ferret badger	2012	KP319198
33	JX12-603	Jiangxi	Ferret badger	2012	KP319199
34	JX12-629	Jiangxi	Ferret badger	2012	KP319200
35	JX12-404	Jiangxi	Ferret badger	2012	KP319214
36	JX12-407	Jiangxi	Ferret badger	2012	KP319215
37	JX12-150	Jiangxi	Ferret badger	2012	KP319228
38	JX12-266	Jiangxi	Ferret badger	2012	KP319229
39	ZJ12-03	Zhejiang	Ferret badger	2012	KP319236
40	JX13-09	Jiangxi	Ferret badger	2013	KP319201
41	JX13-23	Jiangxi	Ferret badger	2013	KP319202
42	JX13-36	Jiangxi	Ferret badger	2013	KP319203
43	JX13-39	Jiangxi	Ferret badger	2013	KP319204
44	JX13-112	Jiangxi	Ferret badger	2013	KP319205

45	JX13-189	Jiangxi	Ferret badger	2013	KP319206
46	JX13-235	Jiangxi	Ferret badger	2013	KP319207
47	JX13-239	Jiangxi	Ferret badger	2013	KP319208
48	JX13-300	Jiangxi	Ferret badger	2013	KP319209
49	JX13-301	Jiangxi	Ferret badger	2013	KP319210
50	JX13-302	Jiangxi	Ferret badger	2013	KP319211
51	JX13-307	Jiangxi	Ferret badger	2013	KP319212
52	JX13-331	Jiangxi	Ferret badger	2013	KP319213
53	JX13-165	Jiangxi	Ferret badger	2013	KP319216
54	JX13-167	Jiangxi	Ferret badger	2013	KP319217
55	JX13-172	Jiangxi	Ferret badger	2013	KP319218
56	JX13-417	Jiangxi	Ferret badger	2013	KP319219
57	JX13-418	Jiangxi	Ferret badger	2013	KP319220
58	JX13-85	Jiangxi	Ferret badger	2013	KP319230
59	JX13-228	Jiangxi	Ferret badger	2013	KP319231
60	JX13-253	Jiangxi	Ferret badger	2013	KP319232
61	JX13-271	Jiangxi	Ferret badger	2013	KP319233
62	JX13-343	Jiangxi	Ferret badger	2013	KP319234
63	JX13-345	Jiangxi	Ferret badger	2013	KP319235
64	ZJ13-66	Zhejiang	Ferret badger	2013	KP319237
65	ZJ13-130	Zhejiang	Ferret badger	2013	KP319238
66	ZJ13-431	Zhejiang	Ferret badger	2013	KP319239
67	JX14-256	Jiangxi	Ferret badger	2014	KX447686
68	JX14-717	Jiangxi	Ferret badger	2014	KX447687
69	JX14-497	Jiangxi	Ferret badger	2014	KX447688
70	JX14-719	Jiangxi	Ferret badger	2014	KX447689
71	JX15-475	Jiangxi	Ferret badger	2015	KY613018
72	JX16-33	Jiangxi	Ferret badger	2016	KY428870
73	JX16-2	Jiangxi	Ferret badger	2016	KY428871
74	JX16-12	Jiangxi	Ferret badger	2016	KY428872
75	JX16-105	Jiangxi	Ferret badger	2016	KY428873
76	JX16-10	Jiangxi	Ferret badger	2016	KY428874
77	JX16-21	Jiangxi	Ferret badger	2016	KY428875
78	JX16-26	Jiangxi	Ferret badger	2016	KY428876
79	JX16-40	Jiangxi	Ferret badger	2016	KY428877
80	JX16-89	Jiangxi	Ferret badger	2016	KY428878
81	JX16-70	Jiangxi	Ferret badger	2016	KY428879
82	JX17-2	Jiangxi	Ferret badger	2017	MT068604
83	JX17-4	Jiangxi	Ferret badger	2017	MT068605
84	JX17-10	Jiangxi	Ferret badger	2017	MT068606
85	JX17-14	Jiangxi	Ferret badger	2017	MT068607
86	JX17-19	Jiangxi	Ferret badger	2017	MT068608
87	JX17-25	Jiangxi	Ferret badger	2017	MT068609
88	JX17-28	Jiangxi	Ferret badger	2017	MT068610
89	JX17-34	Jiangxi	Ferret badger	2017	MT068611
90	JX17-36	Jiangxi	Ferret badger	2017	MT068612
91	JX17-45	Jiangxi	Ferret badger	2017	MT068613
92	JX17-50	Jiangxi	Ferret badger	2017	MT068614
93	JX17-53	Jiangxi	Ferret badger	2017	MT068615
94	JX17-59	Jiangxi	Ferret badger	2017	MT068616
95	JX17-63	Jiangxi	Ferret badger	2017	MT068617
96	JX17-70	Jiangxi	Ferret badger	2017	MT068618
97	JX17-74	Jiangxi	Ferret badger	2017	MT068619
98	JX17-76	Jiangxi	Ferret badger	2017	MT068620
99	JX17-85	Jiangxi	Ferret badger	2017	MT068621
100	JX17-86	Jiangxi	Ferret badger	2017	MT068622

101	JX17-93	Jiangxi	Ferret badger	2017	MT068623
102	JX17-94	Jiangxi	Ferret badger	2017	MT068624
103	JX17-98	Jiangxi	Ferret badger	2017	MT068625
104	JX17-102	Jiangxi	Ferret badger	2017	MT068626
105	JX17-110	Jiangxi	Ferret badger	2017	MT068627
106	JX18-11	Jiangxi	Ferret badger	2018	MT068573
107	JX18-45	Jiangxi	Ferret badger	2018	MT068574
108	JX18-52	Jiangxi	Ferret badger	2018	MT068575
109	JX18-119	Jiangxi	Ferret badger	2018	MT068576
110	JX18-124	Jiangxi	Ferret badger	2018	MT068577
111	JX18-126	Jiangxi	Ferret badger	2018	MT068578
112	JX18-274	Jiangxi	Ferret badger	2018	MT068579
113	ZJF1	Zhejiang	FB	2008	HQ118114
114	HuNPN01	Hunan	Human	2006	DQ496219
115	HuNDN16	Hunan	Dog	2006	DQ515993
116	Guizhou_A10	Guizhou	Dog	2004	DQ666288
117	Guizhou_A101	Guizhou	Dog	2004	DQ666289
118	Guizhou_A103	Guizhou	Dog	2004	DQ666290
119	Guizhou_A148	Guizhou	Dog	2004	DQ666291
120	Henan_Sq30	Henan	Dog	2004	DQ666303
121	Hunan_DK13	Hunan	Dog	2004	DQ666307
122	Hunan_Wg22	Hunan	Dog	2004	DQ666310
123	Hunan_Wg407	Hunan	Dog	2004	DQ666314
124	Jiangsu_Wx0(H)	Jiangsu	Human	2004	DQ666320
125	Jiangsu_Wx1	Jiangsu	Dog	2004	DQ666321
126	Jiangsu_Yc63	Jiangsu	Dog	2004	DQ666322
127	GX01	Guangxi	Dog	2004	DQ866105
128	GX074	Guangxi	Dog	2003	DQ866107
129	GX08	Guangxi	Dog	2003	DQ866108
130	GX091	Guangxi	Dog	2004	DQ866110
131	Zhejiang_Wz0(H)	Zhejiang	Human	2008	EF556197
132	CTN181	Shandong	Vaccine strain	1956	EF564174
133	hubei070308	Hubei	Buffalo	2004	EF611081
134	HNDB11	Hunan	Dog	2005	EU008919
135	Yunnan_Md06	Yunnan	Dog	2006	EU095330
136	FY1	Anhui	Dog	2004	EU159362
137	FY5	Anhui	Dog	2004	EU159365
138	FY9	Anhui	Dog	2004	EU159367
139	FY15	Anhui	Dog	2005	EU159375
140	QC	Hubei	Human	2006	EU159377
141	JSL27	Jiangsu	Dog	2005	EU159382
142	JSL29	Jiangsu	Dog	2005	EU159383
143	Yue1	Guangxi	Dog	1997	EU159385
144	J	Ningxia	Human	1985	EU159387
145	CQ92	Chongqing	Dog	1992	EU159388
146	NC	Jiangxi	Dog	2004	EU159389
147	H69	Anhui	Dog	1969	EU159391
148	SBH	Shanghai	Human	1992	EU159392
149	SBD	Shanghai	Dog	1992	EU159393
150	SH06	Shanghai	Dog	2006	EU159394
151	H	Anhui	Dog	1989	EU159396
152	LH	Zhejiang	Dog	2009	EU159397
153	H89	Anhui	Dog	1989	EU159400
154	WJ	Zhejiang	Dog	2008	EU159401
155	Yunnan_Tc06	Yunnan	Dog	2006	EU275243
156	NeiMeng927A	Inner Mongolia	Raccoon dog	2007	EU284093
157	HN10	Hunan	Human	2006	EU643590

158	NeiMeng927B	Inner Mongolia	Raccoon dog	2007	EU652444
159	Zhejiang_Wz1(H)	Zhejiang	Human	2008	EU700032
160	NeiMeng925	Inner Mongolia	Raccoon dog	2008	FJ415313
161	FJ001	Fujian	Dog	2008	FJ561726
162	FJ003	Fujian	Dog	2008	FJ561728
163	FJ006	Fujian	Dog	2008	FJ561731
164	N11	Guangxi	Dog	1997	FJ594278
165	F02	Zhejiang	FB	2008	FJ712195
166	F04	Zhejiang	FB	2008	FJ712196
167	FJ011	Fujian	Dog	2008	FJ866828
168	FJ012	Fujian	Dog	2008	FJ866829
169	GXLB	Guangxi	Dog	2007	GQ472470
170	Shaanxi-HZ-6	Shaanxi	Dog	2009	GU591790
171	Sichuan-BZ-1	Sichuan	Dog	2009	GU591792
172	CJS0523D	Jiangsu	Dog	2005	HM486348
173	CSH0419D	Shanghai	Dog	2004	HM486356
174	CAH0501D	Anhui	Dog	2008	HM486360
175	CAH0512D	Anhui	Dog	2005	HM486362
176	CJS0621D	Jiangsu	Dog	2006	HM486364
177	CGZ0508D	Guizhou	Dog	2005	HM486368
178	CGZ0620D	Guizhou	Dog	2006	HM486369
179	CSD0801D	Shandong	Dog	2008	HM486376
180	CSD0709D	Shandong	Dog	2007	HM486381
181	HeFei	Anhui	Dog	1989	HQ118104
182	ZJD11	Zhejiang	Dog	2008	HQ118113
183	WQ14-RF	China	Red fox	2014	KM016899
184	CYN1009D	Yunnan	Dog	2012	JQ730682
185	BD06	Hebei	Dog	2006	EU549783
186	TW1680	Taiwan	FB	2013	KF501181
187	Th1749	Taiwan	FB	2013	KP860163
188	TW1907	Taiwan	FB	2013	KP881355
189	Th2170	Taiwan	FB	2013	KP860164
190	Th2229	Taiwan	FB	2013	KP860165
191	Th2284	Taiwan	FB	2013	KP860166
192	Th2408	Taiwan	FB	2013	KP860167
193	Th2484	Taiwan	FB	2013	KP860168
194	Th4957	Taiwan	FB	2013	KP860169
195	Th5114	Taiwan	FB	2013	KP860170
196	TW1682	Taiwan	FB	2013	KF501182
197	TW1683	Taiwan	FB	2013	KF501183
198	Nt1938	Taiwan	FB	2013	KP860149
199	Nt1983	Taiwan	FB	2013	KP860150
200	Nt2169	Taiwan	FB	2013	KP860151
201	Nt2269	Taiwan	FB	2013	KP860152
202	Nt2270	Taiwan	FB	2013	KP860153
203	Nt2274	Taiwan	FB	2013	KP860154
204	TW2700	Taiwan	FB	2013	KP881354
205	Nt2702	Taiwan	FB	2010	KP860155
206	Nt2704	Taiwan	FB	2010	KP860156
207	Nt2706	Taiwan	FB	2012	KP860157
208	Nt2710	Taiwan	FB	2013	KP860158
209	Nt4951	Taiwan	FB	2013	KP860159
210	TW1944	Taiwan	FB	2013	KP881356
211	YL2167	Taiwan	FB	2013	KP860184
212	YL5154	Taiwan	FB	2013	KP860185
213	Cy2143	Taiwan	FB	2013	KP860137
214	Cy2263	Taiwan	FB	2013	KP860138

215	Cy2717	Taiwan	FB	2013	KP860139
216	TW1685	Taiwan	FB	2013	KF501184
217	Th1766	Taiwan	FB	2013	KP860171
218	Th1832	Taiwan	FB	2013	KP860172
219	Th1950	Taiwan	FB	2013	KP860173
220	Th2058	Taiwan	FB	2013	KP860174
221	Th2392	Taiwan	FB	2013	KP860175
222	Th2740	Taiwan	FB	2013	KP860176
223	Th3106	Taiwan	FB	2013	KP860177
224	TW1694	Taiwan	FB	2013	KF501185
225	Kh1710	Taiwan	FB	2013	KP860141
226	Kh1830	Taiwan	FB	2013	KP860142
227	Kh1879	Taiwan	FB	2013	KP860143
228	Kh2261	Taiwan	FB	2013	KP860144
229	Kh2774	Taiwan	FB	2013	KP860145
230	Kh3206	Taiwan	FB	2013	KP860146
231	Kh3597	Taiwan	FB	2013	KP860147
232	Kh3975	Taiwan	FB	2013	KP860148
233	Pt2485	Taiwan	FB	2013	KP860160
234	Pt2946	Taiwan	FB	2013	KP860161
235	Pt3977	Taiwan	FB	2013	KP860162
236	TW1614	Taiwan	FB	2013	KF501180
237	Tt2329	Taiwan	FB	2013	KP860178
238	Tt2514	Taiwan	FB	2013	KP860179
239	Tt4931	Taiwan	FB	2013	KP860180
240	Tt5037	Taiwan	FB	2013	KP860181
241	Tt5040	Taiwan	FB	2013	KP860182
242	Tt5249	Taiwan	FB	2013	KP860183
243	TW1955	Taiwan	FB	2013	KP881353
244	HL1956	Taiwan	FB	2013	KP860189
